# Association of C-reactive protein with osteoarthritis: Evidence from a retrospective analysis

**DOI:** 10.1097/MD.0000000000048835

**Published:** 2026-05-15

**Authors:** Jun Kuang, Weiwei Ma, Cheng Zhang, Zhiyong Hu, Huanan Li

**Affiliations:** a School of Clinical Medicine, Jiangxi University of Chinese Medicine, Nanchang, Jiangxi, China; b Department of Orthopedics, Affiliated Hospital of Jiangxi University of Chinese Medicine, Nanchang, Jiangxi, China.

**Keywords:** C-reactive protein, chronic inflammation, clinical study, mediation effect, osteoarthritis, predictive model

## Abstract

Osteoarthritis (OA) is a common chronic inflammatory joint disease that severely affects the quality of life in middle-aged and older adults. C-reactive protein (CRP) is a classical inflammatory biomarker; however, its cross-sectional association with the presence of OA remains unclear. This study aimed to evaluate the association between CRP and other blood markers with prevalent OA in a hospital-based population. A total of 8652 participants were included in this retrospective cross-sectional study. Multivariable logistic regression models were used to assess the association between CRP and OA, and restricted cubic spline regression was applied to examine dose–response relationships. Subgroup analyses were conducted to explore potential heterogeneity of associations. CRP levels were linearly and positively associated with the presence of OA (*P* < .001), and this association remained statistically significant after multivariable adjustment (odds ratio = 1.013, 95% confidence interval = 1.006–1.021). Restricted cubic spline analysis revealed a linear association between CRP and OA, whereas white blood cell count exhibited a nonlinear pattern. Although statistically significant, the effect size was small, indicating limited clinical relevance at the individual level. In this retrospective cross-sectional study, CRP levels were modestly associated with prevalent OA among hospital-attending adults in China. These findings support an association between systemic inflammation and OA but should be interpreted cautiously given the lack of temporal ordering and potential residual confounding.

## 1. Introduction

Osteoarthritis (OA) is one of the most common chronic diseases among middle-aged and older adults, affecting joint structure and function and significantly reducing individuals’ working capacity and quality of life.^[1]^ Epidemiological data indicate that over 300 million people worldwide suffer from OA, with a disproportionately high incidence and prevalence among middle-aged and elderly populations. With the global rise in aging and obesity rates, the burden of OA is increasing as a major public health concern.^[2]^ Therefore, identifying inflammatory biomarkers associated with OA in clinical populations may help improve the understanding of disease-related inflammatory status, although predictive or causal inferences cannot be made based on cross-sectional data.

Inflammation plays a key role in the onset and progression of OA.^[3,4]^ C-reactive protein (CRP) is a classical acute-phase inflammatory biomarker that reflects a state of low-grade chronic inflammation.^[5]^ Recent studies have suggested that elevated CRP levels are closely associated with an increased risk of various chronic diseases, including cardiovascular disease,^[6]^ chronic obstructive pulmonary disease,^[6]^ and certain autoimmune disorders.^[7]^ However, epidemiological evidence regarding the association between CRP and OA remains inconsistent, particularly due to the scarcity of large-scale cohort studies among middle-aged and older Chinese populations. Moreover, systematic evaluations of the dose–response pattern, subgroup variations, and potential mediating mechanisms in the CRP–OA relationship are still lacking. Based on medical record data from the Affiliated Hospital of Jiangxi University of Chinese Medicine between January 2016 and December 2020, this study aimed to assess the association and dose–response patterns between CRP levels and the presence of OA, and to explore potential subgroup differences, without implying prediction, causation, or disease progression.

## 2. Methods

### 2.1. Study population

**Figure 1.**
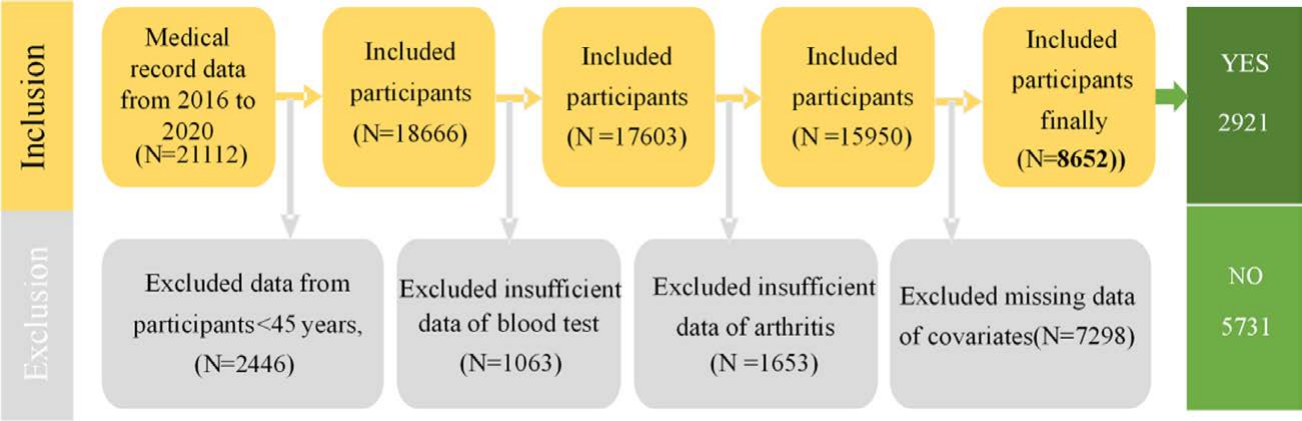
Study population and final participants.

This study was a retrospective analysis, and data were obtained from patients with OA who received treatment at the Affiliated Hospital of Jiangxi University of Chinese Medicine between January 2016 and December 2020. The study population consisted of individuals with complete records in the hospital electronic health system, with an initial screening of 21,112 patients. The following exclusion criteria were applied: age under 45 years or missing age data; lack of relevant blood test data; missing OA (or rheumatism) diagnosis information or follow-up data; incomplete covariate data. Ultimately, 8652 patients were included in the subsequent analysis, of whom 2921 had OA or rheumatism (OA group), and 5731 were normal participants (non-OA group). The screening process is shown in Figure 1. This study was conducted in accordance with the principles of the Declaration of Helsinki and approved by the Ethics Committee of the Affiliated Hospital of Jiangxi University of Chinese Medicine (ethics approval number: JZFYKYLL20250807082).

Note on temporal sequence: this is a retrospective, cross-sectional analysis. The data for exposure (CRP), outcome (OA status), and covariates were extracted from hospital records without a guaranteed temporal sequence. Specifically, for patients with OA, the CRP measurement may have been taken at or after the time of diagnosis. Therefore, the direction of the observed association cannot be conclusively determined. Accordingly, all analyses in this study should be interpreted as descriptive and associative rather than causal.

### 2.2. Assessment of OA

OA was diagnosed according to the clinical and radiographic criteria established by the American College of Rheumatology and the Kellgren–Lawrence grading system. The diagnostic criteria included: persistent joint pain aggravated by activity and relieved by rest, with morning stiffness lasting <30 minutes and functional limitation; physical findings of bony enlargement, joint tenderness, crepitus on motion, and restricted range of motion without marked inflammation; and radiographic evidence of OA defined as Kellgren–Lawrence grade ≥2, characterized by nonuniform joint space narrowing, osteophyte formation, subchondral sclerosis, or cysts. Patients with rheumatoid OA, gout, psoriatic OA, or other traumatic, infectious, and metabolic joint diseases were excluded.

### 2.3. Covariates

This study included numerous potential confounders, such as sociodemographic, socioeconomic variables, health behaviors, and lifestyle factors. Sociodemographic variables included age (years) and sex (male or female). Socioeconomic variables included place of residence (rural or urban), education level (“primary school or below” or “secondary school or above”), and marital status (“married and living together,” “married but separated,” or “other”). Health behaviors and lifestyle factors included smoking status (“nonsmoker” or “smoker”) and drinking status (“nondrinker,” “occasional drinker,” or “frequent drinker”). For smoking status, smokers were defined as individuals who had ever smoked cigarettes, pipes, or cigars. Drinking status was assessed by investigating each participant’s drinking behavior over the past year, including the type of alcohol consumed (e.g., liquor, wine, or beer). Sleep duration was defined as the participant’s average sleep time during the past month. Smoking and drinking status: smoking status was categorized as “smoker” or “nonsmoker.” A “smoker” was defined as an individual who was currently smoking cigarettes, pipes, or cigars at the time of data collection. “Nonsmoker” included both never-smokers and former smokers. Drinking status was categorized as “nondrinker,” “occasional drinker,” or “frequent drinker” based on the participant’s current drinking behavior over the past year. “Nondrinker” included lifelong abstainers and former drinkers. “Occasional drinker” was defined as consuming alcohol on less than (e.g., 1) day per week. “Frequent drinker” was defined as consuming alcohol on (e.g., 1) or more days per week. Due to incomplete or inconsistent documentation in the hospital records, important metabolic comorbidities such as body mass index (BMI), hypertension, and diabetes could not be reliably included in the regression models. Therefore, residual confounding by these factors cannot be excluded and was carefully considered in the interpretation of the results.

### 2.4. Measurement of CRP

Fasting venous blood samples were collected from all participants. Serum CRP levels were measured using a particle-enhanced immunoturbidimetric assay on a Roche Cobas c501 analyzer. The assay used was a high-sensitivity CRP (hs-CRP) assay. The detection range of the assay was 0.2 to 10 mg/L. Values below the lower detection limit were recorded as the lower limit (e.g., 0.2 mg/L), and values above the upper limit were diluted and remeasured according to the manufacturer’s protocol.

### 2.5. Statistical analysis methods

This study aims to investigate the association of blood biomarkers, including CRP, with the risk of OA. The study adopted a retrospective research design and analyzed relevant data from the Affiliated Hospital of Jiangxi University of Chinese Medicine from January 2016 to December 2020. In terms of statistical analysis, if the continuous data met the assumptions of normality and homogeneity of variance, independent *t* tests (for 2 groups) or one-way ANOVA (for 3 or more groups) were applied.^[8]^ For data not meeting these assumptions, nonparametric tests, such as the Mann–Whitney *U* test or Kruskal–Wallis H test, were used. Categorical data were analyzed using the chi-square test.^[9]^ Three logistic regression (LR) models were applied to assess the association between CRP and OA. In addition, restricted cubic spline (RCS) models were used to explore the potential nonlinear relationships between CRP and 4 other blood biomarkers with OA.^[10]^ Three LR models were applied to assess the association between CRP and OA: Model 1 (crude model): no adjustment for covariates. Model 2 (partially adjusted model): adjusted for sociodemographic and socioeconomic variables, including age, sex, place of residence, education level, and marital status. Model 3 (fully adjusted model): adjusted for all variables in Model 2 plus health behaviors and lifestyle factors, including smoking status, drinking status, and sleep duration. The results are presented as odds ratios (ORs) with 95% confidence intervals (CIs). To ensure that our findings were not driven by acute inflammation, we conducted a sensitivity analysis. We excluded all participants with CRP levels >10 mg/L, a conventional cutoff for acute inflammatory states, and repeated the multivariable LR analysis (Model 3) on the remaining cohort (n = 8074).

Further stratified analysis was conducted to evaluate potential interactions between subgroups and CRP. Mediating effects of CRP in the relationship between age and OA were also examined through mediation analysis. All statistical analyses were two-sided, and a *P* value <.05 was considered statistically significant.

## 3. Results

### 3.1. Analysis of general characteristics

**Figure 2.**
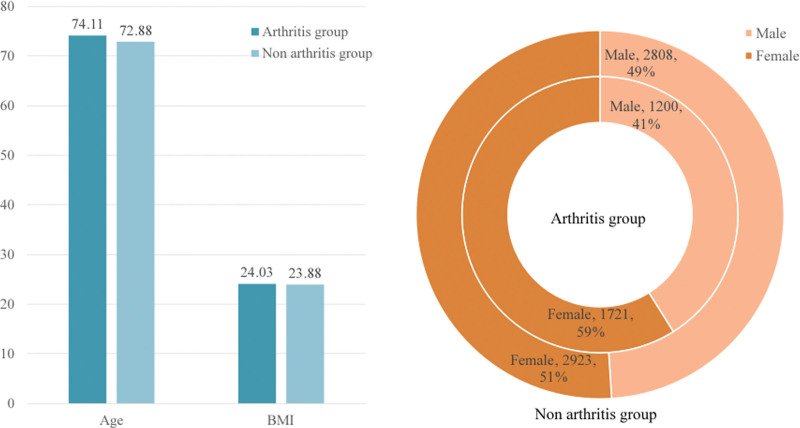
General information of the 2 groups. BMI = body mass index.

The baseline characteristics of the study population are shown in Table 1. A total of 8652 middle-aged and older adults were included, comprising 4008 men (46.3%) and 4644 women (53.7%), among whom 2921 were patients with OA or rheumatism. The mean age of the OA group was 74.11 ± 8.77 years, with an average BMI of 24.03 ± 4.22 kg/m^2^, including 1200 men (41.08%) and 1721 women (58.92%). The non-OA group had a mean age of 72.88 ± 9.05 years and an average BMI of 23.88 ± 4.08 kg/m^2^, consisting of 2808 men (49.00%) and 2923 women (51.00%). The OA group was significantly older than the non-OA group (*P* < .05). Regarding gender, the OA group had a higher proportion of women, and this difference was also statistically significant (*P* < .001). These findings indicate cross-sectional differences between participants with and without OA, which may reflect differences in demographic, lifestyle, and inflammatory characteristics. Figure 2 presents the general characteristics of the 2 groups.

**Table 1:** Baseline characteristics of the study population.

Indicator	Overall	OA group	Non-OA group	*P* value
Number of participants	8652	2921	5731	
Age	73.29 ± 8.97	74.11 ± 8.77	72.88 ± 9.05	<.001
BMI	23.93 ± 4.13	24.03 ± 4.22	23.88 ± 4.08	.18
Gender				
Male	4008	1200	2808	<.001
Female	4644	1721	2923	
Smoking				
Yes	5015	1788	3227	<.001
No	3637	1133	2504	
Drinking				
No	5782	2009	3773	.007
Occasional drinking	677	232	445	
Frequent drinking	2193	680	1513	
Residence				
Urban or other	1973	595	1378	<.001
Rural	6679	2326	4353	
Marital status				
Married and living together	7100	2358	4742	.014
Married but separated	318	102	216	
Others	1234	461	773	
Education level				
Primary school or below	5921	2209	3712	<.001
Secondary school or above	2731	712	2019	
White blood cell	5.97 ± 1.82	5.94 ± 1.90	5.99 ± 1.77	.016
Red blood cell	13.67 ± 1.92	13.53 ± 1.96	13.75 ± 1.89	<.001
C-reactive protein	2.82 ± 6.25	3.19 ± 7.13	2.62 ± 5.74	<.001
Uric acid	4.96 ± 1.41	4.94 ± 1.40	4.96 ± 1.41	.352
Creatinine	0.81 ± 0.28	0.82 ± 0.33	0.81 ± 0.25	.065
Sleep duration	6.39 ± 1.85	6.03 ± 1.95	6.57 ± 1.77	<.001

BMI = body mass index, OA = osteoarthritis.

In terms of socioeconomic variables, the 2 groups differed in the distribution of education level, place of residence, and marital status, with statistically significant differences (*P* < .05). Regarding lifestyle factors, significant differences were also observed between the 2 groups in smoking habits, drinking habits, and sleep duration (*P* < .05). The average sleep duration in the OA group was significantly shorter than that in the non-OA group. These findings suggest that drinking habits, smoking habits, and poor sleep may be potential risk factors for OA. Differences in education level, place of residence, and marital status may also influence the occurrence of OA.

In terms of blood biochemical indicators, except for uric acid and creatinine, significant differences were observed between the 2 groups in white blood cell (WBC) count, hemoglobin, and CRP levels (*P* < .05), indicating that OA may lead to changes in certain blood parameters.

Overall, the study suggests that the occurrence of OA may be closely associated with age, gender, lifestyle factors, socioeconomic status, and changes in certain blood biochemical indicators.

### 3.2. Subgroup analysis

**Figure 3.**
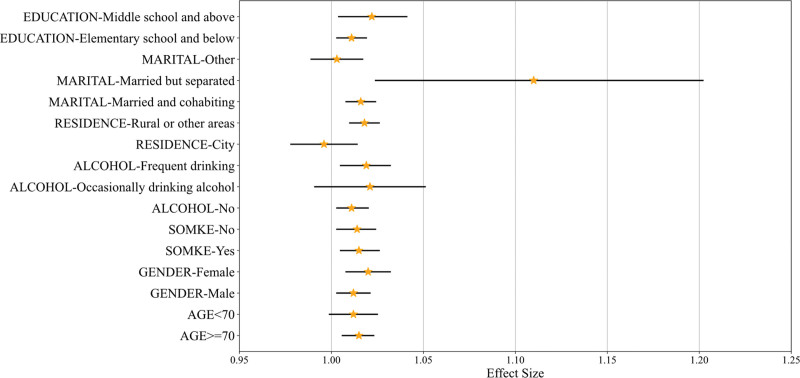
Forest plot of subgroup analyses.

Stratified analyses were conducted based on variables such as age, sex, smoking status, drinking status, place of residence, marital status, and education level. Binary LR analysis was performed to examine the association between CRP and OA events across different subgroups. The results indicated significant interactions between CRP and sex, place of residence, and education level subgroups (*P* for interaction < .001), with statistical significance. In contrast, no significant interactions were found between CRP and age, smoking habits, drinking habits, or marital status (*P* for interaction > .05). Table 2 presents the detailed results of the subgroup analysis, and Figure 3 shows the forest plot of the subgroup analyses.

**Table 2:** Results of subgroup analysis.

Grouping variable	n (%)	OR (95% CI)	*P*	*P* for interaction
Age				
≥70	5550	1.015 (1.006–1.023)	<.001	.992
*<*70	3102	1.012 (0.999–1.025)	.076	
Gender				
Male	4008	1.012 (1.003–1.021)	.008	<.001
Female	4644	1.020 (1.008–1.032)	.001	
Smoking				
Yes	5015	1.015 (1.005–1.026)	.002	.103
No	3637	1.014 (1.003–1.024)	.009	
Drinking				
No	5782	1.011 (1.003–1.020)	.01	.692
Occasional drinking	677	1.021 (0.991–1.051)	.18	
Frequent drinking	2193	1.019 (1.005–1.032)	.007	
Residence				
Urban or other	1973	0.996 (0.978–1.014)	.671	<.001
Rural	6679	1.018 (1.010–1.026)	<.001	
Marital status				
Married and living together	7100	1.016 (1.008–1.024)	<.001	.756
Married but separated	318	1.110 (1.024–1.202)	.011	
Others	1234	1.003 (0.989–1.017)	.702	
Education level				
Primary school or below	5921	1.011 (1.003–1.019)	.005	<.001
Secondary school or above	2731	1.022 (1.004–1.041)	.014	

BMI = body mass index, CI = confidence interval, OA = osteoarthritis, OR = odds ratio.

### 3.3. Logistic regression

To explore the association between CRP and OA, 3 LR models were established. Model 1 did not adjust for any covariates. Model 2 further adjusted for sociodemographic factors (such as age and sex) and socioeconomic variables (such as residence, education, and marital status) based on Model 1. Model 3 additionally adjusted for health behaviors and lifestyle factors (such as smoking, drinking status, and sleep duration) based on Model 2. Table 3 presents the LR results of the models.

**Table 3:** Logistic regression analysis results of each model.

	OR	*P*	95% CI	
			LLCI	ULCI
Model 1	1.014	<.001	1.007	1.021
Model 2	1.013	<.001	1.006	1.02
Model 3	1.013	<.001	1.006	1.021

Model 1 is the initial model without correction of any confounding factors. Model 2 further corrects sociodemographic factors (age and gender) and socioeconomic variables (place of residence, education level, marital status, etc) on the basis of Model 1. On the basis of Model 2, Model 3 further included health behavior and lifestyle factors (such as smoking status, drinking status, and sleep duration) for correction.

CI = confidence interval, LLCI = lower limit confidence interval, OR = odds ratio, ULCI = upper limit confidence interval.

The results showed a significant association between CRP and OA. Specifically, in Model 1 without adjustment, each one-unit increase in CRP concentration was associated with a 1.4% increase in the odds of having OA (OR = 1.014, 95% CI = 1.007–1.021, *P* < .001), which was statistically significant. After adjusting for confounders in Models 2 and 3, the OR values changed slightly, but the relationship between CRP and OA remained consistent with the results of Model 1. Higher CRP levels were associated with higher odds of prevalent OA, although the magnitude of this association was modest (*P* < .001).

### 3.4. Analysis of the association between blood test markers and OA using restricted cubic spline models

**Figure 4.**
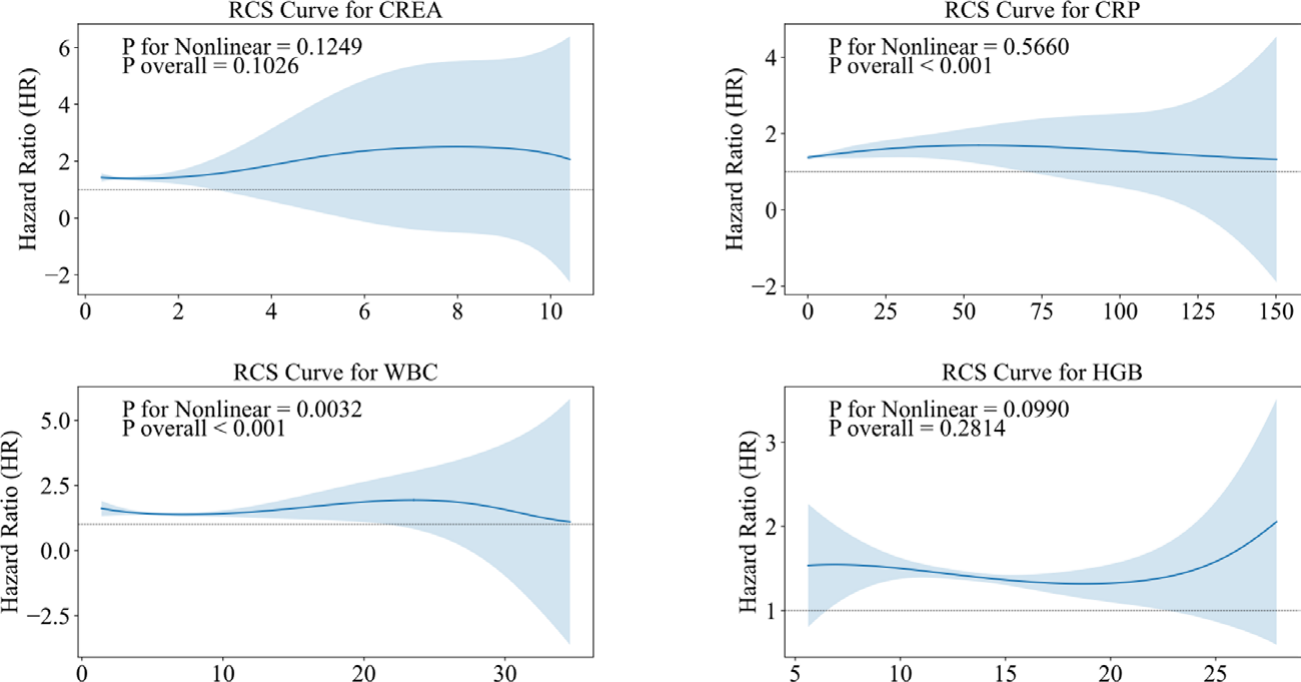
Dose–response relationships between blood test markers and OA risk. CREA = creatinine, CRP = C-reactive protein, HGB = hemoglobin, OA = osteoarthritis, RCS = restricted cubic spline, WBC = white blood cell.

The study used RCS regression to analyze the association between various blood test markers and OA. Figure 4 illustrates the dose–response relationships between 4 blood markers and the risk of OA. The analysis showed that the dose–response relationships between CRP and WBC with OA risk were statistically significant (*P*_overall < .05). CRP exhibited a linear relationship with OA risk (*P* for nonlinear > .05); within the normal CRP range for individuals without acute inflammation or infection, higher CRP levels were linearly associated with an increased risk of OA events. In contrast, WBC displayed a nonlinear relationship with OA risk (*P* for nonlinear < .05).

## 4. Discussion

This result suggests that the relationship between WBC levels and OA risk is not simply linear but shows significant changes or inflection points within certain concentration ranges. In summary, CRP levels showed a linear association with the presence of OA in this cross-sectional analysis. When assessing the health impact of OA, specific distributions of certain blood marker concentrations (such as CRP) and their linear relationships with health outcomes should be considered, especially under high-exposure conditions where greater health risks may exist.

This study systematically investigated the relationship between blood biomarkers, including CRP, and the presence of OA, and examined their cross-sectional associations rather than any predictive or causal effects. It is important to emphasize that our retrospective, cross-sectional design allows us to evaluate associations with prevalent OA but not to establish prediction or causation. The results showed a linear positive association between CRP levels and the presence of OA, which was especially evident in individuals without acute inflammation or infection. This finding indicates that higher CRP levels tend to co-occur with OA in this population, rather than suggesting a temporal or etiological role of CRP. Accordingly, CRP should not be interpreted as a tool for early identification, risk prediction, or screening of OA.

It is important to contextualize the magnitude of the observed association. The odds ratio of 1.013 per 1 mg/L increase in CRP, while statistically significant, is modest from a clinical perspective when considering individual-level differences. This small effect size suggests limited clinical relevance for individual patients and underscores that CRP alone is unlikely to be a clinically meaningful discriminator of OA status. However, this finding can be interpreted descriptively at the population level. First, CRP levels typically vary over a range of several milligrams per liter in the general population. Therefore, a difference of, for example, 3 to 5 mg/L in CRP would correspond to a measurable, although still modest, difference in the odds of prevalent OA. Second, the observed dose–response pattern supports the internal consistency of the association but does not imply biological causation.

Our findings are consistent with previous epidemiological evidence. A seminal meta-analysis by Jin et al^[11]^ demonstrated that circulating CRP levels are higher in individuals with OA than in controls. These prior results, together with our findings, consistently indicate an association between systemic inflammation and OA, but do not establish predictive value or causal pathways. Importantly, although our study involves a large Chinese inpatient population, this does not necessarily enhance generalizability across ethnic groups, as hospital-attending populations may differ substantially from community-based cohorts in disease severity and comorbidity burden.

Numerous clinical studies have reported associations between CRP and OA.^[12,13]^ CRP is an acute-phase protein widely used as a biomarker of systemic inflammation. In patients with OA, elevated CRP levels are often correlated with disease severity and inflammatory activity.^[14]^ Studies have also reported positive associations between serum high-sensitivity CRP (hs-CRP) levels and OA prevalence across subgroups defined by age and sex.^[15]^ In addition, the monomeric form of CRP has been associated with radiographic severity, particularly in advanced OA.^[16]^ These observations suggest that CRP reflects inflammatory burden in OA rather than serving as a diagnostic, predictive, or prognostic marker. Systematic reviews and meta-analyses have similarly shown that hs-CRP levels are higher in OA patients and correlate with pain and functional impairment.^[11]^ A recent meta-analysis by Gao et al^[15]^ further supports this association. Our study adds descriptive epidemiological evidence by demonstrating a linear association using RCS analysis, without implying causality or clinical utility.

From a biological perspective, the observed association between elevated CRP and OA is consistent with the recognized involvement of chronic low-grade inflammation in OA pathophysiology.^[17–19]^ CRP reflects systemic inflammatory activity driven by cytokines such as interleukin-6 and tumor necrosis factor-alpha, which are implicated in synovial inflammation and cartilage degradation.^[20]^ However, these mechanistic considerations remain speculative within the context of this study. Importantly, the cross-sectional nature of our data precludes determination of directionality. It is equally plausible that elevated CRP reflects inflammatory processes secondary to established OA rather than contributing to disease development. Therefore, biological plausibility should not be interpreted as mechanistic evidence.

This study analyzed the association between CRP levels and OA using medical record data from Jiangxi University of Traditional Chinese Medicine Affiliated Hospital from 2016 to 2020, providing observational evidence within a hospital-based population. However, several limitations must be acknowledged. Most importantly, the retrospective cross-sectional design fundamentally limits causal inference and precludes assessment of disease onset, progression, or prediction. Reverse causality remains a key concern. Second, as a single-center hospital-based study, selection bias is likely, and the findings may not be generalizable beyond similar inpatient populations in China. Third, incomplete medical records may have resulted in missing information on disease severity, comorbidities, or medication use. Critically, important confounders such as hypertension, diabetes, and obesity could not be consistently adjusted for due to data limitations. These factors are associated with both CRP and OA and represent a major source of potential residual confounding. Finally, CRP is a nonspecific inflammatory marker influenced by various systemic conditions, which further limits interpretability.

In conclusion, this retrospective cross-sectional study demonstrates a modest association between CRP levels and prevalent OA in a Chinese hospital-attending population. The findings support the notion that systemic inflammation and OA frequently coexist, but do not support claims of prediction, early screening, disease onset, progression, or causal mediation. Future research should prioritize well-designed prospective cohort studies with repeated inflammatory measurements and comprehensive confounder adjustment to clarify temporal relationships. Until such evidence is available, the present findings should be interpreted strictly as descriptive associations rather than clinically actionable evidence.

## Author contributions

**Conceptualization:** Jun Kuang.

**Data curation:** Jun Kuang, Cheng Zhang.

**Formal analysis:** Jun Kuang, Cheng Zhang.

**Resources:** Jun Kuang, Weiwei Ma, Zhiyong Hu.

**Writing – original draft:** Jun Kuang.

**Methodology:** Weiwei Ma.

**Validation:** Weiwei Ma.

**Visualization:** Cheng Zhang.

**Software:** Zhiyong Hu.

**Funding acquisition:** Huanan Li.

**Supervision:** Huanan Li.

**Writing – review & editing:** Huanan Li.
